# Determinants of Implanon discontinuation among women who use Implanon at Debre Berhan town public health institutions in Northeast Ethiopia: a case–control study

**DOI:** 10.3389/fgwh.2023.961364

**Published:** 2023-02-15

**Authors:** Moges Sisay Chekole, Delelegn Tsegaye Zikie, Girma Wogie Fitie, Birhan Tsegaw Taye, Desta Mekete Kibiret, Mulualem Silesh Zerihun, Tebabere Moltot Kitaw, Mohamed Ahmed Ali, Temesegen Desalegn Legasu, Kibir Temesgen Assefa, Tesfanesh Lemma Demisse

**Affiliations:** ^1^ Department of Midwifery, College of Medicine and Health Science, Debre Berhan University, Debre Berhan, Ethiopia; ^2^ Department of Midwifery, College of Medicine and Health Science School of Nursing and Midwifery, Wollo University, Wollo, Ethiopia; ^3^ Department of Midwifery, College of Medicine and Health Science, Metu University, Metu, Ethiopia; ^4^ Department of Midwifery, College of Medicine and Health Science, Jigjiga University, Jigjiga, Ethiopia

**Keywords:** Implanon discontinuation, long-acting reversible contraceptives, Debre Berhan, Ethiopia, case–control

## Abstract

**Background:**

Discontinuation of the most effective methods of contraception such as Implanon has now become a worldwide problem, which is significantly associated with mistimed and unwanted pregnancies and unsafe abortion, leading to an increased risk of maternal and child mortality and morbidity. However, studies on factors associated with Implanon discontinuation in Ethiopia, particularly in the area under this study, are limited. Therefore, this study aims to identify the determinants of Implanon discontinuation among women who used Implanon at Debre Berhan town public health institutions.

**Methods and materials:**

A facility-based unmatched case–control study was conducted among 312 study participants (78 cases and 234 controls) from February 1, 2021, to April 30, 2021. The study participants were selected by using a systematic random sampling method for controls, and cases were selected consecutively until the required sample size was reached, during the data collection period. The data were collected by using a structured face-to-face interviewer-administered questionnaire and entered into Epidata version 4.6 and transferred to SPSS version 25 for analysis. Variables with a *p*-value of <0.25 in the bivariable analysis were entered into the multivariable logistic regression model. In the final model variables, a *p*-value of <0.05 was considered statistically significant at a 95% confidence interval (CI) and the strength of association was measured using an adjusted odds ratio (AOR).

**Results:**

In this study, the determinants of Implanon discontinuation were the following: women who had no formal education (AOR: 3.57; 95% CI, 1.62–7.87), who had no children (AOR: 2.8; 95% CI, 1.50–5.17), who had no counseling about side effects (AOR: 2.43;95% CI, 1.30–4.55), who had no discussion with their partner (AOR: 2.7; 95% CI, 1.34–5.46), who had no follow-up appointment (AOR:2.81; 95% CI, 1.54–5.12), and who had side effects (AOR:1.91; 95% CI, 1.13–3.53).

**Conclusion and Recommendations:**

Women's educational status, having no children during the insertion of Implanon, received no counseling about the side effects of insertion, no follow-up appointment, experiencing side effects, and no discussion with a partner were determinants of Implanon discontinuation. Hence, healthcare providers and other health sector stakeholders should provide and strengthen pre-insertion counseling, and follow-up appointments to increase the retention rates of Implanon.

## 1. Introduction

The implant is a small flexible tube measuring approximately 40 mm in length, which is inserted subdermally in the non-dominant upper arm by trained healthcare providers. Implanon, Jadelle, and Sino-Implant are some of the types of implants (1).

Implanon is a single-rod etonogestrel-containing implant that prevents unintended pregnancy for up to 3 years and immediate restoration of fertility after its removal, with a failure rate of less than 1% (2–5). It prevents pregnancy by inhibiting ovulation, solidifying cervical mucus to prevent the passage of spermatozoa, and altering the lining of the endometrium, which makes it hostile for implantation (6). The insertion and removal of Implanon involve minor surgical techniques; despite this, clients should receive adequate counseling on the effectiveness of the methods, duration of action, possible side effects, and their right to discontinue Implanon use (7). Despite its safety and effectiveness, the discontinuation of the most effective methods of contraception such as Implanon has now become a worldwide problem, which is significantly associated with unintended pregnancies, unwanted birth, and unsafe abortions that lead to an increased risk of pregnancy, childbirth-related maternal morbidity, and deprived infant and child health outcomes (8, 9).

More than 25 million unintended pregnancies happen every year in the world because of the discontinuation of Implanon (9, 10). In Africa, 14 million women become exposed to the risk of unintended conception after 3 months of Implanon discontinuation; for example, more than 40% of women are at risk of conception in Egypt, 51% in Kenya, 73% in Malawi, 47% in Zimbabwe, and 42% in Ethiopia (11). In sub-Saharan Africa (SSA), a large number of women and sexually active adolescents use contraceptive implants, but they discontinue them before the date of appointment, which leads to adverse maternal and child health (MCH) outcomes (12).

According to the Ethiopian Demographic and Health Survey (EDHS) 2016, Ethiopia has a maternal mortality ratio of 412 per 100,000 live births and a total fertility rate of 4.6 children per woman of reproductive age (12). This high mortality and fertility rate underscores the need for paying more attention to providing family planning (FP) service, which helps avoid Implanon discontinuation. The government of Ethiopia plans to increase the contraceptive prevalence rate among married reproductive-age women to 55% and to decrease the unmet need to 10% (13). However, the discontinuation rate of modern contraceptive methods in Ethiopia is 35%, and among them, the implant discontinuation rate within 12 months is 11%. Another study conducted in Debre Markos town shows that 46.5% of women discontinued Implanon early because of poor pre-insertion counseling, service-related dissatisfaction, and absence of postinsertion follow-up (12, 14). In order to reduce the rate of discontinuation of Implanon, the government of Ethiopia has launched an Implanon scale-up initiative designed at escalating access to Implanon and increase the rate of Implanon insertion at the community level by allowing health extension workers to offer Implanon insertion services (13). In addition to this, the Federal Ministry of Health (FMOH) has been collaborating with different partners to advance FP services both at the community and at the facility levels (15). Despite all these efforts at expanding the utilization of Implanon, there is an increasing trend of Implanon discontinuation in Ethiopia nowadays (16).

Existing evidence suggests that experiencing side effects such as irregular and unpredictable vaginal bleeding and also a lack of pre-insertion counseling are among the common risk factors for discontinuation of Implanon (7, 8, 17). There is a contradiction between different studies regarding the factors determining Implanon discontinuation. Previous studies conducted in Ethiopia on such factors were cross-sectional in nature. Therefore, this study intends to identify the determinants of Implanon discontinuation among women who used Implanon in Debre Berhan town public health institutions, Northeast Ethiopia, 2022.

## 2. Materials and methods

### 2.1. Study area and period

A facility-based unmatched case–control study design was conducted at Debre Berhan town public health institutions, located in Amhara Regional State, North Shewa administrative zone. According to information obtained from the District Health Office, in the 2020 population data, the total population size of the district was estimated to be 114,652. Among these people, 62,809 were females and 39,066 were from reproductive age groups. The town is divided into nine urban kebeles and five rural kebeles. There are four governmental health institutions with one referral hospital and three health centers that provide MCH services including family planning. This study was done during the February 1, 2021, to April 30, 2021, period.

### 2.2. Source population

#### 2.2.1. Cases

The cases included all women of reproductive age (15–49 years) who requested Implanon removal in Debre Berhan town public health institutions before the completion of 3 years.

#### 2.2.2. Controls

The controls included women of reproductive age (15–49 years) who requested Implanon removal in the Debre Berhan town public health institutions at the completion of 3 years.

### 2.3. Study population

The study population covered all women of reproductive age who requested Implanon removal in the selected health institutions in the study period.

### 2.4. Eligibility criteria

#### 2.4.1. Inclusion criteria

Women of all child-bearing age groups who were eligible for cases and controls and residing in the study period were included as study participants. On other hand, those who requested the removal of Implanon for conceiving/childbirth during the study period were excluded**.**

### 2.5. Operational definition

#### 2.5.1. Implanon discontinuation

Implanon discontinuation is defined as the discontinuation of the use of Implanon before 3 years following insertion (18).

#### 2.5.2. Counseling

Counseling involves making women aware of the long-term protection of the implant, its side effects, effectiveness, and the advantage of the method (19).

#### 2.5.3. Long-acting reversible contraceptives

These are contraceptive method that serve a period of 3–10 years but can be removed any time (20).

#### 2.5.4. Side Effects

Side effects are the development of at least one of the following conditions: menstrual disruption, headache, weight gain, and insertion site pain (19).

### 2.6. Sample size determination

The sample size for the study was determined using Epi Info version 7.1 software, by considering the following assumptions: the level of significance 95%, power 80%, the ratio of cases to controls 1 : 3, the proportion of controls exposed 28.6%, odds ratio (OR) 2.3, and the percent of cases with exposure 48% from previous similar studies (19, 21). Thus, the minimum adequate sample size for this study was obtained to be 282. By considering a 10% non-response rate, the final sample size turned to be 312 individuals (i.e., 78 cases and 234 controls).

### 2.7. Sampling procedure

The study was conducted at all public health facilities of Debre Berhan town, which include Debre Berhan Referral Hospital, Kebele 04 Health Center, Kebele 08 Health Center, and Kebele 07 Health Center, and all were included in the study. The calculated sample size was proportionally allocated to each health facility on the basis of the previous consecutive 3-month average client flow of the units, which was obtained by referring to client registration logbooks. The average 3-month client flow for Implanon removal in the Debre Berhan Referral Hospital was 30 cases/129 controls, Kebele 04 Health Center 38 cases/142 controls, Kebele 08 Health Center 30 cases/132 controls, and Kebele 07 Health Center 25 cases/97 controls. A total of 123 cases and 500 control women were registered for Implanon removal in each health institution and, among these, the women who removed Implanon for the purposes of conceiving were excluded for sample allocation. In each selected health facility, the cases were selected consecutively until the required sample size was reached, and the controls were selected by using the systematic sampling technique. The control interval for each health facility was 2, and we used the lottery method to select the first client in each health facility on the basis of their sequence of family planning visit.

### 2.8. Data collection tools and procedures

Data were collected using a structured face-to-face interviewer-administered questionnaire adapted from EDHS 2016 and different published literature (14, 21, 22). The questionnaire was first prepared in English and translated to the Amharic version and then back to English to keep its consistency.

It contains three parts: the first contains the sociodemographic characteristics of women, the second consists of the obstetric and gynecologic characteristics of women, and the third method-related characteristics. One Bachelor of Science (BSc) midwife as a supervisor and three diploma midwives were involved in data collection. Both supervisors and data collectors were trained for one day about the purpose of the study, timely collection of data, and overall data collection procedure.

### 2.9. Data processing and analysis

The data were coded and entered using Epidata version 4.6 and exported to the Statistical Package for Social Science (SPSS) version 25 for data checking, cleaning, and analysis. Descriptive statistics including tables and proportions were used to describe the data. Bivariable and multivariable logistic regression analyses were performed to determine the association between outcome and explanatory variables. Variables observed in a bivariable analysis at a *p*-value of <0.25 were considered valid for multivariable logistic regression analysis.

The multicollinearity among independent variables was checked by using the variance inflation factor (VIF) and by considering values that exceeded 10 indicating multicollinearity (in this study: VIF = 1.24), and the goodness-of-fit of the model was checked by using the Hosmer–Lemeshow goodness-of-fit test (i.e., *p* = 0.73 indicated that the model was a good fit). In multivariable logistic regression analysis, a backward likelihood ratio (LR) regression method was used to extract the final model. A significant association was declared for variables with a *p*-value of <0.05 and the strength of the statistical association was determined by using the adjusted odds ratio (AOR) and 95% confidence interval (CI).

## 3. Results

### 3.1. Sociodemographic characteristics of the study participants

A total of 312 individuals (78 cases and 234 controls) took part in the study with a response rate of 98%. The mean age of the case and control women were 26.43 (SD = ±5.569) and 27.67 (SD = ±5.262) years, respectively. In the majority of cases, 69 (88.5%) and three-fourth of controls, 208 (91.2%) belonged to the Orthodox faith. According to the educational status of the participants, 30 (38.5%) cases and 36 (15.9%) controls had no formal education, and 21.8% of cases and 36.8% of controls had college and above level of education. Twenty-seven (34.6%) cases and 71 (31.1%) controls were rural residents and 65.4% of cases and 68.9% of controls were urban residents. With regard to the occupational status of women, 38 (50%) cases and 106 (46.5%) controls were housewives, while 32.9% of cases and 39.8% of controls were employed (Table 1)**.**

**Table 1:** Sociodemographic and economic characteristics of study participants among women who discontinued Implanon in Debre Berhan town, Northeast Ethiopia, 2022.

Variables	Category	Cases (*n* = 78), *n* (%)	Controls (*n* = 228), *n* (%)
Age of respondent in years	<25	31 (39.7)	68 (29.8)
	25–29	29 (37.2)	93 (40.8)
	30–34	6 (7.7)	39 (17.1)
	>35	12 (15.4)	28 (12.3)
Residence of respondents	Urban	51(65.4)	157 (68.9)
	Rural	27 (34.6)	71 (31.1)
Religion of respondents	Orthodox	69 (88.5)	208 (91.2)
	Muslim	7 (9.0)	13 (5.7)
	Others^a^	2 (2.6)	7 (3.4)
Maternal educational level	No formal education	30 (38.5)	36 (15.9)
	Primary education (1–8)	9 (11.5)	32 (14.0)
	Secondary education (9–12)	22 (28.2)	76 (33.3)
	College and above	17 (21.8)	84 (36.8)
Partner's educational status	No formal education	3 (3.9)	8 (3.5)
	Primary education (1–8)	6 (6.5)	22 (9.3)
	Secondary education (9–12)	7 (9.1)	23 (10.1)
	College and above	26 (33.8)	58 (25.6)
		36 (46.8)	117 (51.5)
Maternal occupational status	Housewife	38 (50.0)	106 (46.5)
	Merchant	14 (17.1)	32 (14.0)
	Employed	26 (32.9)	90 (39.5)

^a^ Protestant and Catholic.

### 3.2. Obstetric and gynecological characteristics of study participants

Obstetric history was one of the factors that were assessed in this study. A total of 38 (48.7%) women of the case group and 70 (30.7%) of the control group did not have children during the insertion of Implanon, while 40 women (51.3%) of the case group and 158 (69.3%) of the control group had children. Approximately 32 (41.0%) cases and 142 (62.3%) controls had no history of abortion, while 41% of cases and 62.3% of controls experienced abortion. The purpose or intent of using Implanon among cases 75 (96.2%) and controls 207 (90.8%) was needed to be determined to space out childbirth and that of using others was required to be determined to limit childbirth. With regard to past contraceptive utilization, 25 (32.1%) cases and 98 (43.0%) controls used different types of contraceptives before Implanon insertion. The dominant contraceptives used before insertion were injectable among 22 (28.2%) cases and 90 (39.5%) among controls, followed by implants in 15.4% of cases and 18.1% of controls (Table 2).

**Table 2:** Obstetric and gynecological characteristics of study participants among women who discontinued Implanon in Debre Berhan town, Northeast Ethiopia, 2022.

Variable	Category		Case (*n* = 78), *n* (%)	Controls (*n* = 228), *n* (%)
Having children	Yes		40 (51.3)	158 (69.3)
	No		38 (48.7)	70 (30.7)
No. of children	<4 children		42 (66.7)	118 (75.2)
	≥4 children		21 (33.3)	39 (24.8)
Intent of using Implanon	Spacer		75 (96.2)	207 (90.8)
	Limiter		3 (3.8)	21 (9.2)
History of abortion	Yes		46(59.0)	86 (37.7)
	No		32 (41.0)	142 (62.3
History of contraceptive utilization	Yes		25 (32.1)	98 (43.0)
	No		53 (67.9)	130 (57.0)
Types of contraceptives utilized before Implanon	Pills	Yes	6 (7.7)	42 (18.4)
		No	72 (92.3)	186 (81.6)
	Injectable	Yes	22 (28.2)	90 (39.5)
		No	56 (71.8)	138 (60.5)
	Implant	Yes	12 (15.4)	41 (18.1)
		No	66 (84.6)	185 (81.9)
	IUCD	Yes	3 (3.8)	7 (3.1)
		No	75 (96.2)	221 (96.9)

IUCD, intrauterine device.

### 3.3. Role of the partner and counseling service–related characteristics of study participants

Among those women who used Implanon, 25 (32.1%) cases and 40 (17.5%) controls did not discuss it with their partners before insertion, while 67.9% of cases and 82.5% of controls discussed with their partner. Approximately 46 (59.0%) cases and 126 (55.3%) controls were counseled about the duration of action of Implanon. However, 58 (74.4%) cases and 117 (51.3%) controls were not counseled on the side effects of Implanon. Those who used Implanon by themselves were 51 (65.4%) cases and 147 (64.5%) controls, while 10.3% of cases and 25.4% of controls used Implanon decided by their husbands. Approximately 54 (69.2%) cases and 92 (40.4%) controls did not have any postinsertion follow-up during the Implanon utilization period, but 30.8% of cases and 59.6% of controls had this. A total of 76 (97.4%) cases and 215 (94.3%) controls were implanted by health workers and 2.6% of cases and 5.7% of controls were done so by health extension workers.

In the majority of cases, 54 (69.2%) and 114 (50.0%) controls experienced side effects. Among women who developed side effects, menstrual disruption was by far the most common one for both cases, 46 (59.0%), and controls, 81 (37.5%). The others were amenorrhea 2.6%, headache 28.6%, and weight gain 34.6%. A total of 37 (47.4%) of cases and 58 (25.4%) controls were not satisfied with the services provided during the Implanon insertion period (Table 3).

**Table 3:** Characteristics of study participants regarding counseling and other variables, Debre Berhan, Northeast Ethiopia, 2022.

Variables	Category	Cases (*n* = 78), *n* (%)	Control (*n* = 228), *n* (%)
Counseling about side effects of Implanon	Yes	20 (25.6)	111 (48.7)
	No	58 (74.4)	117 (51.3)
Counseling about advantage	Yes	36 (46.2)	109 (47.8)
	No	42 (53.8)	119 (52.2)
Counseling about effectiveness	Yes	28 (35.9)	85 (37.3)
	No	50 (64.1)	143 (62.7)
Counseling about the duration of action	Yes	46 (59.0)	126 (55.3)
	No	32 (41.0)	102 (44.7)
Types of counseling provided	Individual	18 (60.0)	66 (44.3)
	counseling	9 (30.0)	35 (23.5)
	Mass counseling	7 (23.3)	43 (28.9)
	With partner		
	Counseling		
Discussion with partner	Yes	53 (67.9)	188 (82.5)
	No	25 (32.1)	40 (17.5)
Reason to use Implanon	Together with husband	8 (10.3)	58 (25.4)
	Husband alone	8 (10.3)	14 (6.1)
	Me myself	51 (65.4)	147 (64.5)
	HCP	11 (14.1)	9 (3.9)
Who inserts Implanon	Health extension workers	2 (2.6)	13 (5.7)
	Health workers	76 (97.4)	215 (94.3)
Side effect suffered	Yes	54 (69.2)	114 (50.0)
	No	24 (30.8)	114 (50.0)
Type of side effect suffered?	Menstrual irregularity	46 (59.0)	81(37.5)
	Amenorrhea	2 (2.6)	2 (0.9)
	Headache	22 (28.6)	55 (24.2)
	Weight gain	27 (34.6)	59 (26.0)
Follow-up after insertion	Yes	24 (30.8)	136 (59.6)
	No	54 (69.2)	92 (40.4)
Satisfied with the healthcare provider	Yes	41 (52.6)	170 (74.6)
	No	37 (47.4)	58 (25.4)

### 3.4. Determinants of Implanon discontinuation

Variables with a *p*-value of <0.25 in the bivariable binary logistic regression analysis were considered valid, and therefore, selected for multivariable binary logistic regression analysis. These variables were the following: the participant's educational status, having no children, history of abortion, absence of pre-insertion counseling about side effects, discussion with a partner, experiencing side effects, follow-up appointment given, satisfaction with the service provided, and the reasons for using Implanon. Multivariable binary logistic regression analysis was done to identify the factors that independently determine Implanon discontinuation by controlling the effect of other factors. For example, women with no formal education (AOR: 3.57; 95% CI, 1.62–7.87; *p* = 0.002), those with no children (AOR: 2.8; 95% CI, 1.50–5.17; *p* = 0.001), women who did not receive any pre-insertion counseling about possible side effects (AOR: 2.43;95% CI, 1.30–4.55; *p* = 0.005), those who experienced side effects (AOR:1.91; 95% CI, 1.13–3.53; *p* = 0.039), those who did not have postinsertion follow-up (AOR:2.81; 95% CI, 1.54–5.12; *p* = 0.001), and those who did not discuss with their partners before insertion were all more likely to discontinue Implanon (AOR: 2.7; 95% CI, 1.34–5.46; *p* = 0.006) than those who qualified for the above criteria. History of abortion, service-related dissatisfaction, and the reasons for using Implanon were not statistically associated with Implanon discontinuation in the multivariable binary logistic regression analysis (Table 4).

**Table 4:** Bivariable and multivariable binary logistic regression analyses results indicating the factors associated with the discontinuation of Implanon among women who used the device in Debre Berhan town, Northeast Ethiopia, 2022.

Women's educational status	No formal education	30	36	4.18 (2.021–8.39^a^	3.57 (1.62–7.87)^b^	0.002
	Primary education	9	32	1.39 (.56–3.43)	1.89 (0.693–5.17)	0.213
	Secondary education	22	76	1.43 (.707–2.89)	1.25 (0.569–2.56)	0.575
	College and above	17	84	1	1	
Having children	Yes	40	158	1	1	
	No	38	70	2.14 (1.27–3.63)^a^	2.79 (1.50–5.17)^b^	0.001
History of abortion	Yes	46	86	0.42 (0.249–0.712)*	0.602 (0.327–1.10)	0.602
	No	32	142	1	1	
Counseling about side effects	Yes	20	111	1	1	
	No	58	117	2.75 (1.55–4.86)^a^	2.43 (1.30–4.54)^b^	0.005
Service satisfaction	Yes	41	170	1	1	
	No	37	58	2.65 (1.55–4.52)^a^	1.65 (0.847–3.22)	0.141
Partner discussion	Yes	25	40	1	1	
	No	53	188	2.22 (1.24–3.98)^a^	2.70 (1.34–5.46)^b^	0.006
Experienced side effects	Yes	54	114	2.25 (1.30–3.89)^a^	1.91 (1.14–3.53)^b^	0.039
	No	24	114	1	1	
Postinsertion follow-up	Yes	24	136	1	1	
	No	54	92	3.32 (1.92–5.76)^a^	2.81 (1.54–5.12)^b^	0.001
Reason to use Implanon	Together with husband	8	58	0.113 (0.036–3.56)^a^	0.308 (0.79–1.19)	0.89
	Husband alone	8	14	0.468 (0.136–1.61)	0.484 (0.101–2.32)	0.25
	Me myself	51	147	0.284 (0.111–0.724)	0.39 (0.13–1.20)	0.103
	Healthcare provider	11	9	1	1	

^a^ Candidate variables for multivariable analysis at a *p*-value of <0.25.

^b^ Statistically significant at *p* < 0.05 in multivariable logistic regression; 1 = reference category.

## 4. Discussion

This study aimed to identify the determinants of Implanon discontinuation and address the most relevant factors resulting in such discontinuation in Debre Berhan town public health institutions, Northeast Ethiopia.

In the multivariable logistic regression model, the women's educational status, having no children during Implanon insertion, a lack of pre-insertion counseling about the possible side effects of Implanon, the absence of follow-up appointment, no discussion with a partner before insertion, and experiencing the side effects of insertion were all significantly associated with Implanon discontinuation.

The study participants who had no formal education were significantly associated with the discontinuation of Implanon. Those women who did not have any formal education were 3.57 times more likely to discontinue Implanon than those whose educational levels were college and above. This finding is in line with that of studies done in Duguna Fango Woreda Wolayita Zone, Southern Ethiopia (21), Durame town, southern Ethiopia (23), Bangladesh (24), and Ilorin, Nigeria (25), which conclude that the discontinuation of Implanon is affected by the educational status of Implanon users. This might be due to the fact that women with no formal education may not have the necessary autonomy to decide on contraceptive issues and may have less knowledge and awareness of long-acting contraceptive methods including Implanon. Also, rumors and misconceptions about long-acting contraceptive methods may lead to discontinuation. In contrast, women who attain high levels of education may not discontinue Implanon because they may have a better understanding of the information from service providers or others when side effects occur (26). However, this finding is different from that of a study done in Debre Markos, Ethiopia (14). This variance might be attributed to the difference in the demographic characteristics of the participants, who include both urban and rural residents, in this study.

This study also found that women who did not have any children during the insertion of Implanon were 2.8 times more likely to discontinue their Implanon than those who had children. This finding is consistent with that of a study done in Bahir Dar town, northeast Ethiopia (19) and Debre Tabor town of Amhara region (28). The rates of discontinuation that vary according to the category of women could be attributed to many factors. For example, women who have had children would have received family planning counseling in each cascade of intervention during pregnancy or continuum of care, which ultimately results in the women having good knowledge or exposure to family planning information many times than those who have not had children. On the other hand, those who have had no children are mostly young nulliparous women who have had less exposure to family planning counseling and less experience in family planning involving adverse or known side effects of the methods used than those who have had children.

In this study, it was found that women who did not receive pre-insertion counseling about side effects were determinants of Implanon discontinuation. The odds of Implanon discontinuation among women who did not receive such counseling were 2.43 times more than those who were counseled. This finding is consistent with that of studies done in Egypt (19), Kenya (27) Mekelle city in Tigray region (18), and Dale District, southern Ethiopia (28). Women who are properly counseled on the possible side effects of pre-Implanon insertion will tolerate the side effects much better than their non-counseled counterparts during and after the procedure. Contraceptive counseling has a great role to play in empowering women who have no desire for pregnancy to choose birth control methods that the women use correctly and consistently over time, thereby reducing the risks of unintended pregnancy and unsafe abortion (29). Those who do not discuss with their partner are 2.7 times more likely to discontinue Implanon than those who do. This finding is similar to that of a study done in Bahir Dar town in northeast Ethiopia (19). Incorporating the male partner in family planning or contraceptive service utilization programs has an important role to play in increasing contraceptive continuation (30). Not having discussions with the partner may give rise to a conflict of interest with the partner in terms of having additional children, which may lead to discontinuation. In addition, the partner might not accept sexual disturbances caused by menstrual irregularity, which is secondary to the side effects of Implanon. Another reason might also be social pressures in favor of maintaining high fertility and rumors about the risks of future sterility that warrant early insertion removal for nulliparous women. In this study, another major determinant factor that was found to be significantly associated with Implanon discontinuation was women not having any appointments for follow-up after insertion.

The odds of Implanon discontinuation among women who did not have any postinsertion follow-up were 2.8 times more likely than those who did so. This finding is in agreement with that of a study done in Dale district, southern Ethiopia (28), Bahir Dar town (19), and Debre Tabor town, Amhara Region (22). The advantage of follow-up is that clients can have additional information (counseling) about Implanon insertion and receive supportive treatment from healthcare providers for any possible side effects. Another possible advantage is that during follow-up service, women might get proper information about any concerns or misconceptions regarding the use of methods that lead to Implanon discontinuation.

In this study, it was found that those women who experienced possible side effects of Implanon were 1.91 times more likely to discontinue insertion than those who did not. This finding was consistent with that of studies conducted in South Africa (7), Andabet District in Southwest Ethiopia (31), and Kinshasa city of the Democratic Republic of Congo (4). The reason for discontinuation might also be related to ineffective and insufficient counseling and information services on the side effects of Implanon. The other reason might be women who develop irregular vaginal bleeding may disturb the partner's sexual feelings and feel guilty about such normal occurrence of bleeding. Those who are not appropriately informed about the possible side effects during counseling may be troubled and worried by unexpected changes in their mood, menstruation patterns, and weight (26).

### 4.1. Implication of results on the target group

In this study, the determinants of Implanon discontinuation were as follows: women with no formal education, those who did not receive pre-insertion counseling about the possible side effects of Implanon, and those who did not have any follow-up appointments. Therefore, it is important for health sector stakeholders and healthcare providers to provide appropriate counseling to their clients about the possible side effects of pre-Implanon insertion and proper follow-up postinsertion. Healthcare authorities and other relevant bodies such as the Ministry of Education should also create awareness among those women who have no formal education and their partners by dissemination of information, education, and communication in order to increase the retention rates of Implanon.

### 4.2. Limitations of the study

Due to the nature of the study design, there might be recall bias and social desirability bias, and hence, the study did not include the factors related to the health system and healthcare providers.

## 5. Conclusion

The determinants of Implanon discontinuation in this study were as follows: women's educational status, absence of children, absence of discussion with a partner, lack of pre-insertion counseling about the possible side effects, experiencing side effects, and absence of follow-up appointments. Therefore, healthcare providers and other health sector stakeholders should provide appropriate and effective pre-insertion counseling and follow-up appointments to their clients in order to increase the retention rates of Implanon.

## Data Availability

The original contributions presented in the study are included in the article/Supplementary Material, further inquiries can be directed to the corresponding author.
